# Health associations of various fruit forms: solid fruits, juices, and smoothies

**DOI:** 10.3389/fnut.2025.1626179

**Published:** 2026-04-08

**Authors:** Shubhreet Bhullar

**Affiliations:** University of California, Los Angeles, Los Angeles, CA, United States

**Keywords:** fruit, juice, smoothie, health outcomes, public health, dietary recommendations, non-communicable diseases (NCDs)

## Abstract

**Introduction:**

The rising prevalence of non-communicable diseases (NCDs) highlights the need for refined dietary recommendations that distinguish between forms of fruit consumption. This study examines the associations between solid fruits, fruit juices, and fruit smoothies with various health outcomes.

**Methods:**

A cross-sectional survey (*N* = 443) collected self-reported dietary, health, and demographic data, categorizing participants into four groups: low fruit consumers, fruit juice consumers, solid fruit consumers, and fruit smoothie consumers.

**Results:**

Results revealed notable differences in health outcomes. The fruit smoothies group demonstrated the best overall health, with the lowest hypertension prevalence (18.60%), lowest frequency of mental health struggles (12.79%), and highest self-rated energy levels (4.51 ± 0.70). In contrast, the fruit juices group had the poorest outcomes, including the highest high cholesterol prevalence (39.18%) and lowest self-rated sleep quality (2.95 ± 0.85). Low fruit consumers had the highest cardiovascular disease rates (16.17%) and most physician visits (5.48 ± 1.01).

**Discussion:**

While self-reported data and potential confounders pose limitations, findings underscore the need to differentiate fruit consumption types in dietary guidelines. Retaining fiber while enhancing nutrient bioavailability, fruit smoothies may offer unique health benefits. Future longitudinal studies are needed to establish causality and refine dietary recommendations for public health.

## Introduction

A healthy diet is crucial in preventing non-communicable diseases (NCDs) like heart disease, diabetes, and cancer, as unhealthy eating habits (high sugar, high saturated fat, high salt, low fruit, and low vegetable consumption) contribute to the development of NCDs (1). Identifying and promoting effective dietary patterns is crucial for improving public health due to the ability of a healthy diet to improve overall health and longevity while reducing healthcare costs (2). Although a substantial number of studies have examined various aspects of diet ranging from vegetables and meat to sugar and fat, certain food groups — particularly fruits — are often overlooked. Fruits serve as excellent sources of essential nutrients, fiber, and antioxidants, which can contribute to overall health and wellbeing by supporting digestion, boosting the immune system, and protecting against chronic diseases (3). However, most studies analyze fruit and vegetable consumption rather than just focusing on fruits, leaving the relationship between fruits and health outcomes in question (4). For example, a systematic review analyzing fruit and vegetable intake performed a meta-analysis of 142 studies, 24 of which focused solely on fruit consumption (5). However, all of these studies focused on fruit consumption as a whole and failed to consider that there are various unique methods of ingesting fruits, such as drinking fruit juices, drinking fruit smoothies, or eating solid fruits.

Solid fruits — whole, raw, or minimally processed (i.e., sliced/chopped) — are traditionally considered to be the best way to consume fruits, although dietary guidelines insist that all fruit consumption forms are equal (6). Moreover, many believe 100% fruit juices (juice derived solely from fruits) and fruit smoothies (blended fruits) to be equally nutritious as solid fruits, which is consistent with dietary guidelines (7). The validity of such beliefs must be assessed via scientific research. Fruit juice is the liquid extracted from a fruit while a fruit smoothie is the blended beverage made from the entire fruit and retains fiber, pulp, and other solids. This difference in juicing vs. blending is believed to cause variation in the respective glycemic indices of fruit juices and fruit smoothies. However, as an umbrella review study shows, there is a limited number of studies examining multiple fruit consumption forms. An umbrella review study, which found decreased incidence of cardiovascular disease, stroke, congenital heart disease, and oral cancer, examined 77 meta-analyses — of which only one was regarding an alternate fruit consumption form to solid fruits or fruits in general (8).

In addition, there is an ongoing scientific debate regarding the efficacy of fruit juices, with some studies linking them to metabolic issues and increased incidence of diabetes/hyperglycemia and other studies showing positive effects. While some studies have found an increase in cardiovascular disease mortality associated with fruit juice consumption (9), others found fruit juices to correlate with decreased cancer, hypertension, and cardiovascular disease incidence (10). Furthermore, there is a paucity of research comparing the different fruit consumption types, particularly fruit smoothies, on various measures of health. For example, the only studies examining the health outcomes of fruit smoothies focus on one or two specific types of fruits (e.g., raspberries, nutraceutical fruits) or on one specific health outcome (e.g., psychological distress) (11). Additionally, public health campaigns and dietary guidelines often overlook misconceptions about fruit juices, treating all forms of fruit equally despite evidence that processing alters nutrient composition and glycemic response, potentially leading to overconsumption (12).

This study aims to refine dietary recommendations by examining how different forms of fruit consumption — solid fruits, fruit juices, and fruit smoothies — uniquely impact various health outcomes, including chronic diseases, mental health, sleep quality, energy levels, and medical resource use. While prior research has established the benefits of fruit consumption, it often generalizes all forms of fruit as equally beneficial, failing to consider how processing may influence nutrient composition and health effects. By employing a survey-based methodology, this study provides a more comprehensive analysis of physical and mental health factors, lifestyle habits, and medical usage, addressing gaps in prior research that typically focus on limited outcomes like cardiovascular disease or body mass index (BMI). The findings could enhance dietary guidelines by promoting specific fruit consumption patterns for optimal health and cognitive function. Given the potential public health implications, this study underscores the importance of differentiating between fruit consumption types to guide more precise nutritional recommendations. The following methods section details the study’s design, participant selection, and analytical approach, ensuring a thorough investigation of these associations.

## Materials and methods

### Study design and participants

This research paper employed a cross-sectional, survey-based study to investigate possible correlations between different forms of fruit consumption (solid fruits, fruit juices, and fruit smoothies) and various health outcomes. A convenience sample of 443 adults aged 18–65 years was recruited from urban centers in the Greater Sacramento area in California. This includes El Dorado, Nevada, Placer, Sacramento, Sutter, Yolo, and Yuba counties. These participants were approached in public spaces such as marketplaces, local community centers, and university campuses to gather a diverse sample of individuals. Participants were excluded from the study if they did not fit the age range of 18–65, if they had pre-existing chronic illness unrelated to the study variables (e.g., cancer, autoimmune diseases, and so forth), if they were pregnant or breastfeeding, or if they had medically prescribed or highly restrictive diets (e.g., ketogenic, vegan, and so on. Individual with a history of substance abuse or regular use of recreational drugs (at least once a month) were also excluded to minimize the effect of confounding factors on the health outcomes of this study’s participants. The survey was administered orally to the participants and responses were recorded directly into a designated Google Sheet, allowing for efficient data entry and easy organization.

The target sample size of approximately 440 participants was chosen because it balances feasibility with the need for reasonably sized subgroups. With group counts ranging from 86 to 167 participants, each fruit-consumption category was large enough to capture natural variability within the population, avoid extremely small cells, and ensure that estimates within each group were stable rather than driven by a few outliers. In studies relying on in-person, community-based sampling, achieving group sizes above ∼75–100 per category is generally sufficient for meaningful descriptive and exploratory comparisons, while remaining realistic. The final enrollment of 443 participants met these qualitative benchmarks and provided adequate representation across all fruit-consumption groups.

Institutional Review Board (IRB) approval was not required for this study, since no identifying information was taken, the participants remained anonymous, no invasive interventions were done, the study was completely optional, and all participants expressed informed consent prior to participation. The International Healthcare Organization’s Institutional Review Board granted this study exempt determination from IRB approval on these grounds.

### Survey design

The survey questionnaire was designed to record demographic information, fruit consumption habits, and health outcomes. Basic demographics such as age, gender (sex assigned at birth), and highest level of educational attainment (high school diploma, bachelor’s degree, graduate degree, or none of the above) were obtained. In addition to this, the researcher recorded whether the participants exercise regularly (exercise for a total of at least 1.5 h a week), are smokers (smoke at least once a week), use alcohol (consume alcohol at least once a week), and use drugs (consume non-prescription drugs recreationally, as in less than once a month). Participants were also asked to identify their self-perceived socioeconomic status as either low, medium, or high based on their relative income, lifestyle, and net worth. Personal information regarding finances was not collected; participants were instructed to quietly evaluate and state either low, medium, or high.

Participants reported which type of fruit (solid, juice, or smoothie) they consume with the greatest frequency based on their dietary habits in the past year. Solid fruits were defined as either whole or sliced fruits consumed in their natural solid form. Fruit juices were defined as the liquid extracted from solid fruits, including store-bought and homemade juices. Fruit smoothies were defined as liquid blends of solid fruits, including store-bought and homemade smoothies. The fruit juices and fruit smoothies had to have been 100% derived from fruits and could not contain added sugar, preservatives, or colorings. Smoothies with additives like yogurt, milk, or ice cream were not considered fruit smoothies. Participants were divided into four groups based on whether they consumed more solid fruits, fruit juice, fruit smoothies, or were low fruit consumers (those who, on average, consumed less than one serving of fruit in any form per day). This low fruit consumers group serves as a control for comparison with the health outcomes of the different types of fruit consumers. A further exclusion criterion was set for those who reported consuming nearly equal amounts (within 10% range in servings) of two or more fruit forms (solid, juice, smoothie). This exclusion was set in place to ensure that participants were unambiguously sorted into one of the four consumption groups. This not only created a clear, logical framework for categorization, but also allowed for a fair representation of typical dietary habits and allowed the researcher to assess the primary dietary influence on health outcomes without overcomplicating the analysis with overlapping groups.

The health outcomes variables of the survey were designed to measure the overall health of the survey respondents without performing any invasive interventions. The number of physician visits/medical appointments in the last year was self-reported by each participant of each consumption group and averaged to receive a separate mean and standard deviation for each group. Participants were told to self-report diagnosis or past history of hypertension, high cholesterol, diabetes, high blood sugar, and cardiovascular disease. Past history is included because it acknowledges the long-term impact of such conditions that may influence current health or have dietary triggers/associations even if the condition is no longer active. Participants declared whether they experienced mental health struggles or used prescription medications for chronic (non-communicable) conditions within the past year. The BMI of the participants was calculated during the survey administration from weight and height measurements using a portal weighing machine and a measuring tape. The mean BMI and standard deviation for each consumption group was calculated from the aforementioned data. Participants provided a self- assessment of their overall health in the past year on a scale of 1–5 (1 being very poor and 5 being excellent), the frequency and severity of chronic musculoskeletal pain in the past year on a scale of 1–5 (1 being no pain and 5 being severe, frequent pain), average sleep quality over the past month on a scale of 1–5 (1 being very poor and 5 being excellent), and average daily energy levels over the past month on a scale of 1–5 (1 being very low and 5 being very high). Musculoskeletal pain, defined as pain affecting bones, muscles, ligaments, tendons, or joints, aligns more directly with the potential effects of fruit consumption (such as anti-inflammatory properties that, in turn, contribute to increased immune function). Despite the fact that self- perceived health is a strong predictor of healthcare utilization and long-term health outcomes, it is often excluded from quantitative studies (13). Incorporating self-reported perceptions into dietary research can enhance our understanding of how individuals experience the health impacts of their dietary choices, improving the practical relevance of dietary guidelines.

This survey was pilot-tested on a smaller sample of 24 individuals to refine the survey, ensuring clarity, relevance, and participant engagement. Due to the extreme discomfort of some participants in disclosing their ethnicity, the researcher chose to exclude ethnicity from the final survey design to prioritize participant comfort and encourage honest reporting on health, dietary, and demographic factors. In addition to this, pilot testing enabled the refinement of the assignment criteria for the four consumption groups (as well as the creation of the low fruit consumers group) and those who consumed nearly equal amounts of two or more different fruit forms. The pilot-testing also resulted in the refinement of time frames for the self-assessed health outcomes and the inclusion of key variables like self-reported high cholesterol and blood sugar.

## Results

### Demographic characteristics of participants

**FIGURE 1:**
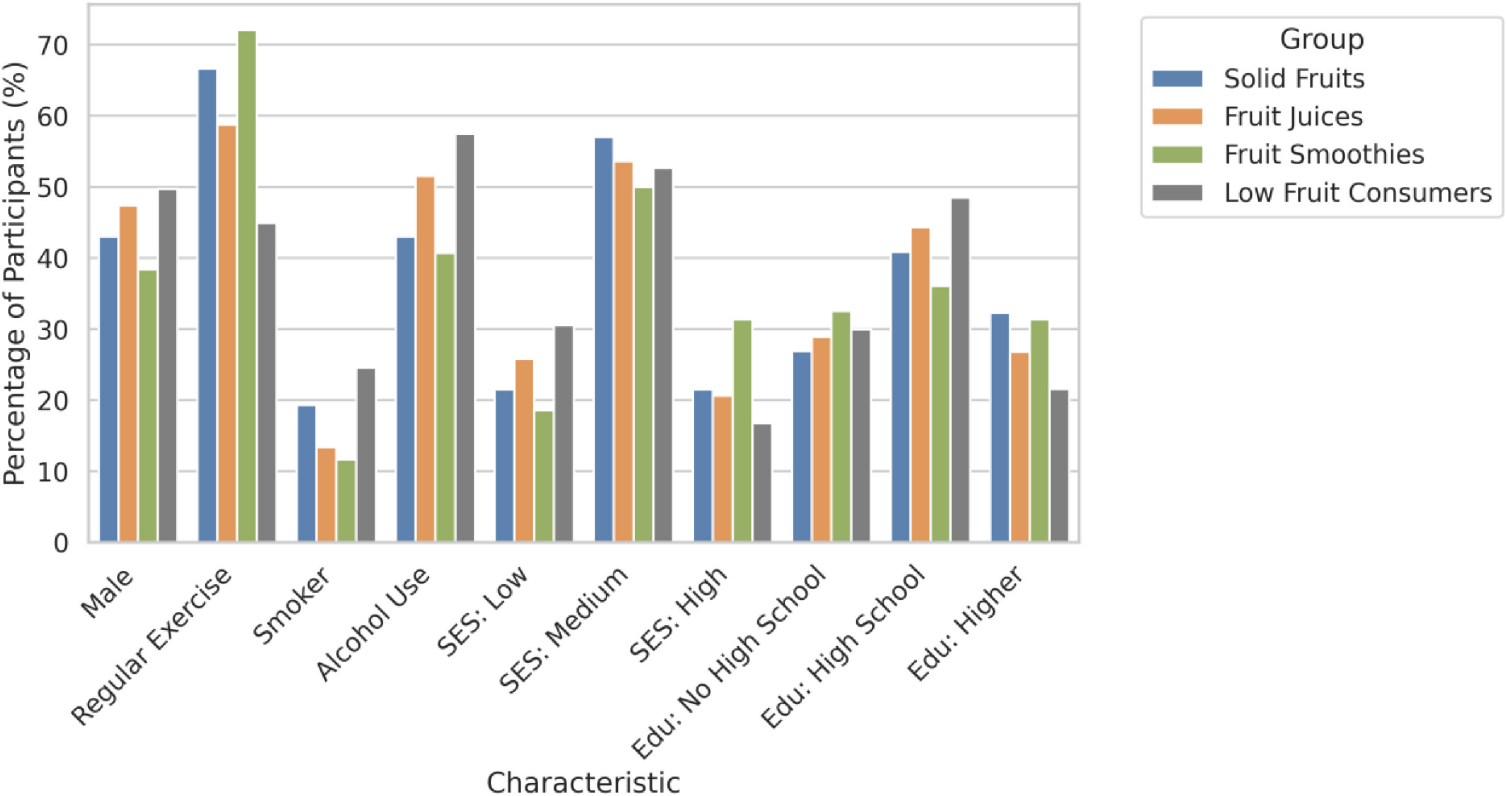
Demographic characteristics by fruit consumption group.

As seen in Table 1, the demographic characteristics (age, gender, socioeconomic status, educational attainment, exercise habits, smoker status, alcohol use, and recreational drug use) were collected and summarized for each fruit consumption group. Of the 443 study respondents, 93 fell into the solid fruits group, 97 into fruit juices, 86 into fruit smoothies, and 167 into low fruit consumers. The mean age, gender percentages, levels of educational attainment, and socioeconomic status did not vary much between the four groups. The majority of participants across all groups fell into the medium socioeconomic status category (56.99%, 53.61%, 50.00%, and 52.69%, respectively). Across the solid fruits, fruit juices, fruit smoothies, and low fruit consumers groups, 32.26%, 26.81%, 31.40%, and 21.56% of participants had college degrees, respectively. Regarding the frequency of exercisers, smokers, alcohol users, and recreational drug users, the solid fruits group had 66.67%, 19.35%, 43.01%, and 9.68%, respectively; meanwhile, the fruit juices group had 58.76%, 13.40%, 51.55%, and 12.37%, respectively. The fruit smoothies group had the highest frequency of regular exercisers (72.09%) and the lowest frequency of smokers (11.63%), alcohol users (40.70%), and recreational drug users (8.14%). The low fruit consumers group had the lowest frequency of regular exercisers (44.91%) and the highest frequency of smokers (24.55%), alcohol users (57.49%), and recreational drug users (19.76%). The key characteristics for each group are displayed in Figure 1.

**TABLE 1:** Demographic information of participants.

Demographic characteristic	Solid fruits (*n* = 93)	Fruit juices (*n* = 97)	Fruit smoothies (*n* = 86)	Low fruit consumers (*n* = 167)
Age (mean ± SD)	35.42 ± 12.17	37.89 ± 13.06	33.75 ± 11.24	40.11 ± 14.72
Gender				
Male (%)	43.01% (*n* = 40)	47.42% (*n* = 46)	38.37% (*n* = 33)	49.70% (*n* = 83)
Female (%)	56.99% (*n* = 53)	52.58% (*n* = 51)	61.63% (*n* = 53)	50.30% (*n* = 84)
Socioeconomic status				
Low (%)	21.51% (*n* = 20)	25.77% (*n* = 25)	18.60% (*n* = 16)	30.54% (*n* = 51)
Medium (%)	56.99% (*n* = 53)	53.61% (*n* = 52)	50.00% (*n* = 43)	52.69% (*n* = 88)
High (%)	21.51% (*n* = 20)	20.62% (*n* = 20)	31.40% (*n* = 27)	16.77% (*n* = 28)
Educational attainment				
No high school diploma (%)	26.88% (*n* = 25)	28.87% (*n* = 28)	32.56% (*n* = 28)	29.94% (*n* = 50)
High school diploma (%)	40.86% (*n* = 38)	44.33% (*n* = 43)	36.05% (*n* = 31)	48.50% (*n* = 81)
Bachelor’s degree (%)	22.58% (*n* = 21)	18.56% (*n* = 18)	24.42% (*n* = 21)	15.57% (*n* = 26)
Graduate degree (%)	9.68% (*n* = 9)	8.25% (*n* = 8)	6.98% (*n* = 6)	5.99% (*n* = 10)
Regular exercise (%)	66.67% (*n* = 62)	58.76% (*n* = 57)	72.09% (*n* = 62)	44.91% (*n* = 75)
Smoker (%)	19.35% (*n* = 18)	13.40% (*n* = 13)	11.63% (*n* = 10)	24.55% (*n* = 41)
Alcohol use (%)	43.01% (*n* = 40)	51.55% (*n* = 50)	40.70% (*n* = 35)	57.49% (*n* = 96)
Recreational drug use (%)	9.68% (*n* = 9)	12.37% (*n* = 12)	8.14% (*n* = 7)	19.76% (*n* = 33)

This data table includes basic demographic information, ranging from age and gender to socioeconomic status and educational attainment, in addition to some other factors included to discern differences between the four fruit consumption groups.

### Mean number of physician visits in the past year

As Table 2 depicts, participants who were low fruit consumers (*n* = 167) had an average of 5.48 ± 1.01 physician visits in the past year, while those who consumed solid fruits (*n* = 93), fruit juices (*n* = 97), and fruit smoothies (*n* = 86) had an average of 3.12 ± 0.73, 4.91 ± 0.89, and 2.84 ± 0.62, respectively.

**TABLE 2:** Health outcomes of participants.

Health outcome variable	Solid fruits (*n* = 93)	Fruit juices (*n* = 97)	Fruit smoothies (*n* = 86)	Low fruit consumers (*n* = 167)
Mean number of physician visits (per year)	3.12 ± 0.73	4.91 ± 0.89	2.84 ± 0.62	5.48 ± 1.01
Hypertension (%)	21.51%	32.99%	18.60%	40.72%
Self-reported high cholesterol (%)	19.35%	39.18%	20.93%	19.16%
Diabetes (%)	10.75%	58.76%	8.14%	11.98%
Self-reported high blood sugar (%)	62.37%	54.64%	70.93%	48.50%
Cardiovascular disease (%)	6.45%	13.40%	3.49%	16.17%
Mental health struggles (%)	16.13%	22.68%	12.79%	30.54%
Prescription medication use (%)	35.48%	61.86%	23.26%	52.10%
Mean BMI	23.87 ± 2.83	28.36 ± 3.57	22.71 ± 2.64	26.42 ± 3.22
Average self-assessment of overall health (1–5)	4.16 ± 0.78	3.72 ± 0.84	4.54 ± 0.71	3.14 ± 0.91
Average self-assessment of musculoskeletal pain (1–5)	2.67 ± 0.65	3.01 ± 0.72	2.48 ± 0.58	3.39 ± 0.85
Average self-assessment of sleep quality (1–5)	4.32 ± 0.80	2.95 ± 0.85	4.08 ± 0.73	3.43 ± 0.88
Average self-assessment of energy levels (1–5)	4.21 ± 0.76	2.68 ± 0.81	4.51 ± 0.70	3.19 ± 0.89

This data includes medical resource access, prevalence of chronic conditions/non-communicable diseases (NCDs), prevalence of mental health struggles, body mass index (BMI), and self-assessments of general health indicators. These health outcomes are used to analyze the health trends between the four different fruit consumption groups.

### Prevalence of chronic conditions among participants

In regard to hypertension, the solid fruits, fruit juices, fruit smoothies, and low fruit consumers groups had respective prevalences of 21.51%, 32.99%, 18.60%, and 40.72%. Meanwhile, these groups had respective prevalences of high cholesterol of 19.35%, 39.18%, 20.93%, and 19.16%; respective prevalences of diabetes of 10.75%, 58.76%, 8.14%, and 11.98%; and respective prevalences of high blood sugar of 62.37%, 54.64%, 70.93%, and 48.50%.

### Mental health struggles reported by participants

While 16.13% of the solid fruits group and 12.79% of the fruit smoothies group experienced mental health struggles within the past year, this number was much higher in the fruit juices (22.68%) and low fruit consumers (30.54%) group.

### Prescription medication use among participants

The majority of the members of the fruit juices (61.86%) and low fruit consumers (52.10%) groups have used prescription medications in the past year for non-communicable conditions. The solid fruits group (35.48%) has a moderate amount of prescription medication users, while the fruit smoothies group (23.26%) has the least amount.

### Mean BMI of participants

The mean body mass index (BMI) of the fruit consumption groups in ascending order is as follows: fruit smoothies (22.71 ± 2.64), solid fruits (23.87 ± 2.83), low fruit consumers (26.42 ± 2.64), fruit juices (28.36 ± 3.57).

### Self-assessments of various general health indicators

**FIGURE 2:**
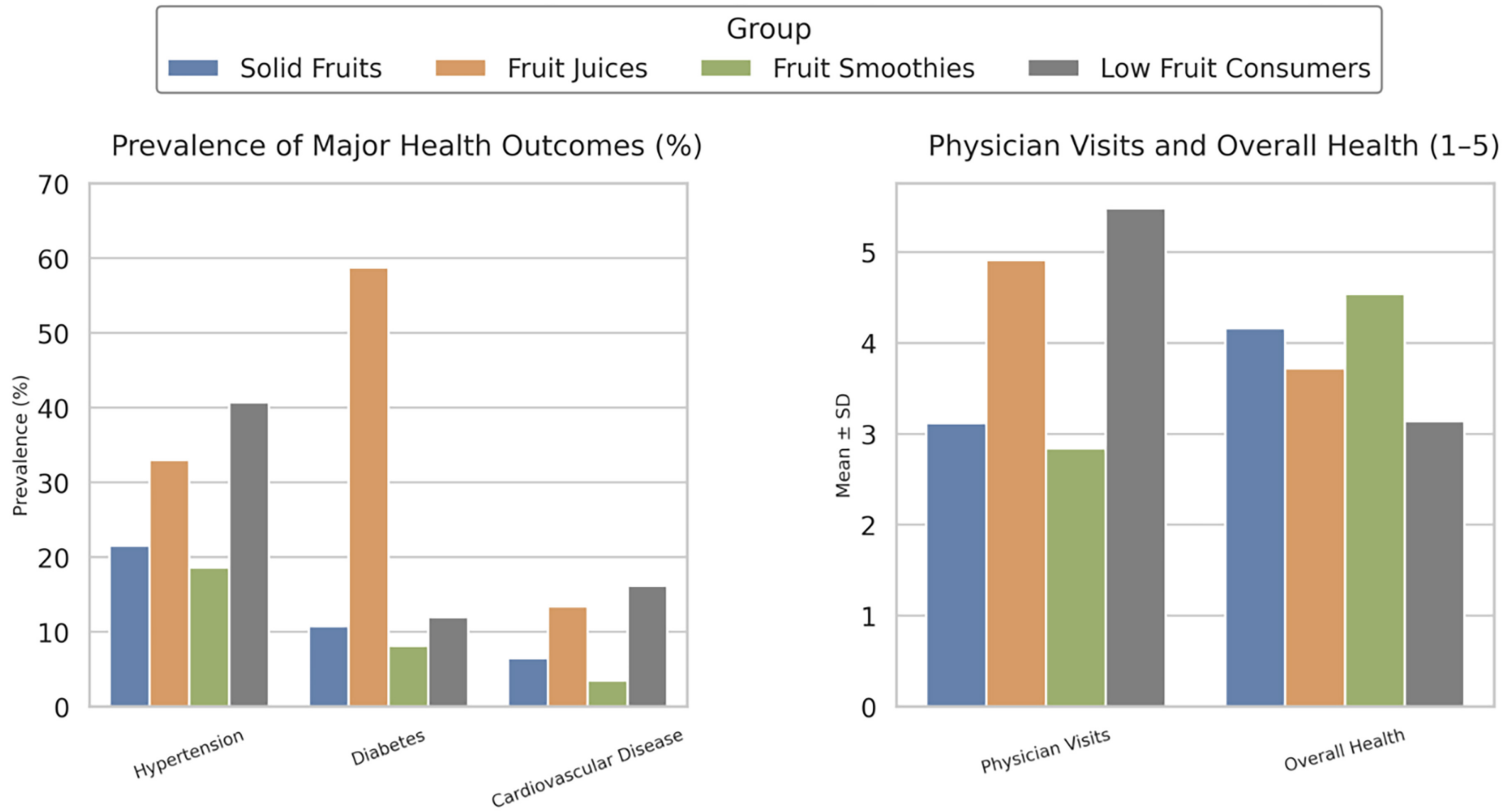
Key health outcomes by fruit consumption group.

Regarding overall health, the fruit smoothies group (4.54 ± 0.71) self-assessed themselves as the highest on average, followed by solid fruits (4.16 ± 0.78), then fruit juices (3.72 ± 0.84), and then low fruit consumers (3.14 ± 0.91). The fruit smoothies group (2.48 ± 0.58) had the least musculoskeletal pain, followed by the solid fruits group (2.67 ± 0.65), then the fruit juices group (3.01 ± 0.72), and then the low fruit consumers group (3.39 ± 0.85). The fruit juices group had the worst sleep quality (2.95 ± 0.85), and energy levels (2.68 ± 0.81), while the low fruit consumers group had the second worst (3.43 ± 0.88 and 3.19 ± 0.89, respectively). The fruit smoothies group had the highest energy levels (4.51 ± 0.70) and the second-best sleep quality (4.08 ± 0.73), while the solid fruits group had the best sleep quality (4.32 ± 0.80) and the second highest energy levels (4.21 ± 0.76). The key findings can be visualized in Figure 2.

### Regression analysis

To account for potential confounders, multivariable regression models were used with low fruit consumers as the reference group. The results of these models are presented in Table 3.

**TABLE 3:** Multivariable-adjusted associations between predominant fruit form consumption and health outcomes, using low fruit consumers as the reference group.

Health outcome variable	Group	Adjusted estimate (95% CI)	*P*-value
Hypertension	Solid fruits	0.57 (0.29–1.12)	0.101
	Fruit juices	1.63 (0.85–3.14)	0.138
	Fruit smoothies	0.42 (0.18–0.96)	0.039*
High cholesterol	Solid fruits	1.08 (0.53–2.22)	0.832
	Fruit juices	2.59 (1.31–5.11)	0.006**
	Fruit smoothies	1.01 (0.42–2.44)	0.983
Diabetes	Solid fruits	0.95 (0.37–2.41)	0.913
	Fruit juices	14.60 (7.16–29.77)	<0.001***
	Fruit smoothies	0.64 (0.20–2.03)	0.445
High blood sugar	Solid fruits	1.23 (0.73–2.07)	0.436
	Fruit juices	1.36 (0.81–2.27)	0.243
	Fruit smoothies	2.10 (0.81–2.27)	0.011*
Cardiovascular disease	Solid fruits	0.38 (0.13–1.14)	0.085
	Fruit juices	0.83 (0.36–1.94)	0.671
	Fruit smoothies	0.19 (0.05–0.72)	0.015*
Mental health struggles	Solid fruits	0.41 (0.22–0.78)	0.007**
	Fruit juices	0.59 (0.32–1.09)	0.089
	Fruit smoothies	0.29 (0.13–0.66)	0.003**
Prescription medication use	Solid fruits	0.54 (0.29–1.01)	0.052
	Fruit juices	2.54 (1.37–4.71)	0.003**
	Fruit smoothies	0.39 (0.18–0.85)	0.018*
Physician visits (mean)	Solid fruits	−1.79 (−2.39 to −1.19)	<0.001***
	Fruit juices	−0.87 (−1.47 to −0.26)	0.005**
	Fruit smoothies	−2.29 (−2.91 to −1.67)	<0.001***
Mean BMI	Solid fruits	−2.74 (−3.68 to −1.79)	<0.001***
	Fruit juices	0.91 (−0.09 to 1.91)	0.076
	Fruit smoothies	−3.82 (−4.82 to −2.82)	<0.001***
Overall health (1–5)	Solid fruits	0.76 (0.45 to 1.07)	<0.001***
	Fruit juices	0.13 (−0.18 to 0.44)	0.404
	Fruit smoothies	1.30 (0.97 to 1.64)	<0.001***
Musculoskeletal pain (1–5)	Solid fruits	−0.62 (−0.94 to −0.31)	<0.001***
	Fruit juices	−0.45 (−0.77 to −0.13)	0.006**
	Fruit smoothies	−0.87 (−1.20 to −0.54)	<0.001***
Sleep quality (1–5)	Solid fruits	0.85 (0.54–1.16)	<0.001***
	Fruit juices	−0.74 (−1.06 to −0.42)	<0.001***
	Fruit smoothies	0.63 (0.29–0.96)	<0.001***
Energy level (1–5)	Solid fruits	0.90 (0.59–1.22)	<0.001***
	Fruit juices	−0.80 (−1.12 to −0.47)	<0.001***
	Fruit smoothies	1.39 (1.05–1.73)	<0.001***

Values are adjusted odds ratios (aOR) with 95% confidence intervals for binary outcomes (hypertension, high cholesterol, diabetes, high blood sugar, cardiovascular disease, mental health struggles, prescription medication used) and adjusted β coefficients with 95% confidence intervals for continuous outcomes [physician visits, body mass index (BMI), overall health, musculoskeletal pain, sleep quality, and energy level]. Logistic and linear regression models were adjusted for age, sex, socioeconomic status, education, physical activity, smoking, alcohol use, and drug use. Models for non-BMI continuous outcomes were additionally adjusted for BMI. A *p*-value less than 0.05 (*p* < 0.05) was considered statistically significant.

*Indicates statistical significance at *p* < 0.05 (statistically significant).

** Indicates statistical significance at *p* < 0.01 (statistically highly significant).

***Indicates statistical significance at *p* < 0.001 (statistically very highly significant).

For chronic conditions, fruit juice consumption was strongly associated with higher odds of diabetes (aOR 14.60, 95% CI 7.16–29.77, *p* < 0.001) and prescription medication use (aOR 2.54, 95% CI 1.37–4.71, *p* = 0.003), while smoothie intake was associated with lower odds of hypertension (aOR 0.42, 95% CI 0.18–0.96, *p* = 0.039), cardiovascular disease (aOR 0.19, 95% CI 0.05–0.72, *p* = 0.015), mental health struggles (aOR 0.29, 95% CI 0.13–0.66, *p* = 0.003), and prescription medication use (aOR 0.39, 95% CI 0.18–0.85, *p* = 0.018). Solid fruit consumers showed modest but favorable associations, including reduced odds of mental health struggles (aOR 0.41, 95% CI 0.22–0.78, *p* = 0.007).

For continuous health indicators, solid fruit and smoothie consumers reported significantly lower BMI compared with low fruit consumers (β = −2.74, 95% CI −3.68 to −1.79, *p* < 0.001; β = −3.82, 95% CI −4.82 to −2.82, *p* < 0.001, respectively). Smoothie consumption was also associated with fewer physician visits (β = −2.29, 95% CI −2.91 to −1.67, *p* < 0.001), higher overall self-rated health (β = 1.30, 95% CI 0.97–1.64, *p* < 0.001), lower musculoskeletal pain (β = −0.87, 95% CI −1.20 to −0.54, *p* < 0.001), better sleep quality (β = 0.63, 95% CI 0.29–0.96, *p* < 0.001), and higher energy levels (β = 1.39, 95% CI 1.05–1.73, *p* < 0.001). Juice consumers, but contrast, demonstrated significantly lower sleep quality (β = −0.74, 95% CI −1.06 to −0.42, *p* < 0.001) and energy (β = −0.80, 95% CI −1.12 to −0.47, *p* < 0.001).

Taken together, these adjusted findings indicate that solid fruits and smoothies are associated with more favorable health profiles across multiple outcomes, whereas fruit juices, despite being a common source of fruit intake, showed detrimental association, particularly with diabetes and overall vitality.

## Discussion

As previously mentioned, this study applies a novel approach by examining the different forms of fruit consumption (solid fruits, fruit juices, fruit smoothies) and their associations with a wide variety of health outcomes ranging from chronic/lifestyle conditions to self-assessments of personal health. Given the knowledge that a healthy lifestyle (healthy dietary patterns and physical activity) can prevent many non-communicable diseases (NCDs), it is important to clearly identify the most effective dietary patterns in order to minimize the risk of developing NCDs (14). The overarching goal of this study is to inspire more focused causal research in the field of dietary healthy, while also promoting the increased specificity of dietary guidelines, such as clearly elucidating the distinctions between fruit consumption forms.

Prior to analyzing the data and compiling the results of this study, it was hypothesized that solid fruits would be the healthiest option, with fruit smoothies and fruit juices being the second and third best options, respectively. Fruit smoothies and fruit juices have lower energy intakes than solid fruits, suggesting that they might not be as beneficial (12, 15). However, this was not quite the case, as the fruit smoothies group was associated with the best health outcomes.

The key findings of our study reveal that the low fruit consumers group had the worst health outcomes, while the fruit juices group had the second worst. The fruit smoothies group had the lowest percentage of prescription medication use (23.26%), while the fruit juices group had the most (61.86%). The fruit juices group had the highest percentage of high cholesterol (39.18%) and second highest percentage of hypertension (32.99%). The fruit smoothies group had the lowest prevalence of hypertension (18.60%), while the low fruit consumers group (40.72%) had the highest. The low fruit consumers and fruit juices groups had the highest (16.17%) and second highest (13.40%) prevalence of cardiovascular diseases, respectively.

As seen in a longitudinal analysis study, low fruit consumption and high fruit juice consumption are both correlated with all-cause mortality in women with cardiovascular diseases (16). Nonetheless, a systematic review study found no correlation between fruit juice consumption and coronary heart disease or diabetes, despite finding that 100% fruit juice consumption resulted in decreased blood pressure (17). Additionally, this study reported a non-linear dose-response relationship between 100% fruit juice consumption and decreased risk of stroke and cardiovascular events (17). Conversely, authors found the consumption of high excess free fructose beverages (such as fruit drinks and juices) to be associated with increased prevalence of coronary heart disease (18). However, these researchers failed to differentiate between 100% fruit juices, fruit drinks that include added sugars or preservatives, and soft drinks. The congregation of fruit consumption types with varying levels of postulated healthiness, combined with the focus on merely a couple of health outcome variables, reveals the necessity of a research paper with clearly defined fruit consumption groups and numerous diverse health outcomes.

Expanding upon these sundry health outcomes, our study reveals that the fruit smoothies group and solid fruits groups had the lowest (12.79%) and second lowest (16.13%) frequency of mental health struggles, respectively. While past studies have shown that raw fruits and vegetables impact mental health more positively than processed ones (19), they lacked a comparison with a blended smoothie form of fruits (20). Furthermore, even though the fruit juices group had the highest prevalence of diabetes by far (58.76%), the fruit smoothies group had the highest percentage of people with high blood sugar (70.93%), followed by the solid fruits group (62.37%). Although fruit juices lack the fiber that solid fruits possess and therefore have a higher glycemic index, fruit smoothies still possess this fiber. Glycemic index (GI) is a scale used to rank carbohydrate-containing foods based on how quickly they raise blood sugar levels after consumption, with a lower score being more favorable. However, the blending of fruits into smoothies breaks down the fruits’ cellular structures (21), usually, but not always, leading to a lower glycemic index according to the results of various studies (22). For example, although apples, berries, and passionfruit showed a decreased GI in smoothie form as compared to their solid form (21, 23), mangos showed no difference, potentially due to their high sugar content relative to other fruits (24). Considering the disagreements between previous literature regarding the relative glycemic indices of solid fruits and fruit smoothies, this study’s results regarding diabetes and high blood sugar might not be extremely generalizable.

Besides high blood sugar and high cholesterol, the fruit smoothies group had the lowest prevalence of chronic conditions, as well as the lowest mean BMI and physician visits. In addition to this, the fruit smoothies group had the lowest musculoskeletal pain (2.48 ± 0.58) and the highest energy levels (4.51 ± 0.70). The low fruit consumers group has the highest musculoskeletal pain (3.39 ± 0.85). While fruit consumption has not been causally linked to muscle recovery (25), it has been shown to decrease lower back pain (26).

The fruit juices group had the lowest energy levels (2.68 ± 0.81) and the worst sleep quality (2.95 ± 0.85), which is consistent with the fact that fruit juices provide the least energy and satiety of the fruit consumption forms (27). The solid fruits group had the best sleep quality (4.32 ± 0.80), keeping in line with the notion that fruit consumption two hours before bedtime promotes sleep (27).

Moreover, the order of average self-assessed overall health from least to greatest in the four fruit consumption groups was as follows: low fruit consumers (3.14 ± 0.91), fruit juices (3.72 ± 0.84), solid fruits (4.16 ± 0.78), fruit smoothies (4.54 ± 0.71). Interestingly, the average overall health rankings of the groups align precisely with their relative healthiness, as indicated by the observed health outcomes. A previous research study revealed the fruit smoothies can have increased nutrient bioavailability compared to solid fruits, as the breaking of cell walls via blending enables the body to absorb nutrients more efficiently (21). In addition to this, a fruit smoothie of a mix of fruits has a lower glycemic index than that same mix of fruits in their solid form (24). These factors may help explain why the fruit smoothies group exhibited the most favorable overall health profile. By retaining the fiber naturally present in solid fruits while also enhancing access to polyphenols, carotenoids, and other bioactive compounds, smoothies may promote satiety, improve postprandial glucose response, and support cardiometabolic health more effectively than juices (28–30). Blending may also facilitate the release of micronutrients such as vitamin C and folate, which contribute to antioxidant activity and overall metabolic function (31, 32). Additionally, individuals who regularly consume fruit smoothies may be more health-conscious in general, which could partially contribute to the observed associations (33). While fruit smoothies are correlated with better health outcomes in this study, as well as some other research studies, the desire to follow healthy dietary and lifestyle patterns (which is correlated with fruit smoothie consumption) could be a potential confounding variable (24).

Relating these findings to current dietary recommendations revealed both consistencies and notable gaps in existing guidance. The DASH (Dietary Approaches to Stop Hypertension) Diet, developed to promote cardiovascular health, emphasizes increased fruit intake and reduced consumption of sugar-sweetened beverages (34). However, it does not clearly distinguish between solid fruits, (100%) fruit juices, and fruit smoothies, instead focusing on eliminating artificially sweetened beverages (35, 36). In our study, individuals in the fruit juice group exhibited the highest incidence of high cholesterol and the second-highest rates of cardiovascular disease and hypertension, whereas previous research has reported that moderate 100% fruit juice consumption may reduce blood pressure and lower stroke risk (17). The DASH Diet provides examples only of solid fruits, and although some clinicians and dietitians incorporate fruit smoothies within its framework, empirical evidence supporting this inclusion is limited (37). Similarly, the Dietary Guidelines for Americans, 2020–2025 do not explicitly reference fruit smoothies, though they highlight the favorable nutritional profile of solid fruits compared to fruit juices (38–40). While our findings generally align with these recommendations, the observed adverse outcomes associated with fruit juice consumption and the scarcity of research on smoothies emphasize the need for clearer, evidence-based distinctions among fruit consumption forms in future dietary guidelines.

While performing experiments to elucidate causality between variables is important, correlational studies provide suggestions for potential causations, enabling researchers to efficiently narrow down their experiments. Correlational studies, like this one, are unable to control for confounding variables, like physical activity, overall diet quality, and socioeconomic status. However, because this study uses a cross-sectional design, temporality can not be established, and therefore no causal inferences can be drawn. This also raises the possibility of reverse causation (for example, individuals with existing health conditions may alter their fruit-form choices) as well as unmeasured or residual confounding even after statistical adjustment. Another potential source of error in this study is the reliance upon self-reported data, which is inherently subject to inaccuracies such as recall bias (misremembering information) or social desirability bias (providing answers they believe to be socially acceptable) that might impact the data collected for demographics, fruit consumption, or health outcomes. These self-reported measures may contribute to both random and systematic errors in exposure and outcome classification. Additionally, biomarkers or objective health indicators were not measured in this study, potentially limiting the accuracy of the findings. Furthermore, a larger and more diverse participant pool would have provided a more representative analysis of the population, strengthening the generalizability of the results. Because recruitment occurred in public urban settings, the sample reflects convenience sampling, which may introduce selection bias and limit external validity. Another limitation of this study is the lack of quantification of fruit consumption within the various fruit consumption groups, which might have provided novel revelations. Quantifying servings or grams per day would have enabled evaluation of potential dose-response relationships. The survey also did not collect information on the specific fruit types included in each category. Since fruits differ substantially in fiber content, phytochemical composition, glycemic index, and micronutrient density, variations in fruit selection could contribute to the health patterns observed. Future research should distinguish not only between fruit forms but also between the predominant fruits used within each category. Despite these limitations, this research provides useful descriptive and associative insights into fruit-form consumption patterns and highlights the need for longitudinal and experimental studies to more definitively explore these relationships.

Currently, as aforementioned, dietary recommendations do not detail the negative health effects associated with fruit juice consumption that are detailed in this study, and they often do not differentiate between various forms of fruit (solid, juice, smoothie). Also, many research studies do not consider fruit smoothies when discussing the impacts of fruits on health, which, considering this study’s revelation that fruit smoothie consumption is correlated with the best health outcomes, is a severe hinderance to the understanding of dietary science. Furthermore, given the incredible diversity among humans, research exploring tailored dietary recommendations for curtailing the risk of chronic diseases is promising. It is also important for studies to examine other dietary factors, such as the co-consumption of food with fruits, the quantity of fruit consumption, and the quality of the fruit consumed. Longitudinal studies that include biomarker measurements for health outcomes are needed for the assessment of causal relationships. This paper lays a foundation for future studies intended to boost our dietary understanding and promote better overall public health.

## Data Availability

The raw data supporting the conclusions of this article will be made available by the authors, without undue reservation.
